# Serum concentrations of Krebs von den Lungen-6, surfactant protein D, and matrix metalloproteinase-2 as diagnostic biomarkers in patients with asbestosis and silicosis: a case–control study

**DOI:** 10.1186/s12890-017-0489-0

**Published:** 2017-11-17

**Authors:** Changjiang Xue, Na Wu, Xue Li, Meihua Qiu, Xuqin Du, Qiao Ye

**Affiliations:** 1 Department of Occupational Medicine and Toxicology, Clinical Center for Interstitial Lung Diseases, Beijing Chao-Yang Hospital, Capital Medical University, 8 Gongren Tiyuchang Nanlu, Chao-Yang District, Beijing, 100020 China; 2 Department of Pathology, Beijing Chao-Yang Hospital, Capital Medical University, 8 Gongren Tiyuchang Nanlu, Chao-Yang District, Beijing, 100020 China

**Keywords:** Asbestosis, Silicosis, Biomarker, Krebs von den Lungen-6, Surfactant protein D, Matrix metalloproteinase, Pulmonary function

## Abstract

**Background:**

Asbestosis and silicosis are progressive pneumoconioses characterized by interstitial fibrosis following exposure to asbestos or silica dust. We evaluated the potential diagnostic biomarkers for these diseases.

**Methods:**

The serum concentrations of Krebs von den Lungen-6 (KL-6), surfactant protein D (SP-D), and matrix metalloproteinase-2 (MMP-2), MMP-7, and MMP-9 were measured in 43 patients with asbestosis, 45 patients with silicosis, 40 dust-exposed workers (DEWs) without pneumoconiosis, and 45 healthy controls (HCs). Chest high-resolution computed tomography (HRCT) images were reviewed by experts blinded to the clinical data. According to the receiver operating characteristic (ROC) curve, the ideal level of each biomarker and its diagnostic sensitivity were obtained.

**Results:**

The serum KL-6 and MMP-2 concentrations were highest in patients with asbestosis, particularly in comparison with those in DEWs and HCs (*P*<0.05). The serum SP-D concentration was significantly higher in patients with asbestosis than in patients with silicosis, DEWs, and HCs (*P*<0.01), whereas no significant difference was noted among patients with silicosis, DEWs, and HCs. No significant difference in the serum MMP-7 or -9 concentration was found among patients with asbestosis, patients with silicosis, DEWs, or HCs. Among patients with asbestosis, the serum KL-6 concentration was significantly correlated with the lung fibrosis scores on HRCT and negatively correlated with the forced vital capacity (FVC) % predicted and diffusing capacity of the lung for carbon monoxide (DL_CO_) % predicted. The serum SP-D and MMP-2 concentrations were negatively correlated with the DL_CO_ % predicted (all *P*<0.05). The order of diagnostic accuracy according to the ROC curve was KL-6, SP-D, and MMP-2 in patients with asbestosis alone and in the combination of both patients with asbestosis and those with silicosis. The combination of all three biomarkers may increase the possibility of diagnosing asbestosis (sensitivity, 93%; specificity, 57%) and both asbestosis and silicosis (sensitivity, 83%; specificity, 62%).

**Conclusions:**

KL-6, SP-D, and MMP-2 are available biomarkers for the adjuvant diagnosis of asbestosis and silicosis. The combination of all three biomarkers may improve the diagnostic sensitivity for asbestosis and silicosis.

## Background

Asbestosis and silicosis are progressive pneumoconioses characterized by interstitial fibrosis following exposure to asbestos or silica dust. Disease progression usually leads to irreversible complications and death. Clinical detection and diagnosis of asbestosis and silicosis mainly relies on a history of occupational exposure and radiological abnormalities [1, 2]. China, where the asbestos and silica exposure industries have not been greatly limited, has invariably exhibited a sustained epidemic of asbestosis and silicosis [3, 4]. Biomarkers with which to detect these pneumoconioses other than imaging and lung function testing are warranted [5]. Krebs von den Lungen 6 (KL-6), surfactant protein D (SP-D), and matrix metalloproteinases (MMPs) are potential biomarkers for diagnosing and monitoring progression of various fibrotic lung diseases.

KL-6, classified in humans as mucin, is a circulating high-molecular-weight glycoprotein [6]. KL-6 has served as a useful biomarker in the differential diagnosis of various chronic lung fibroses and pulmonary alveolar proteinosis, evaluation of disease activity, and prediction of disease outcome [6–10]. The regenerating type II pneumocytes can be positively stained by the KL-6 monoclonal antibody in the lung tissue of patients with lung fibrosis [6, 7]. KL-6 is detectable in both the serum and bronchoalveolar lavage fluid of patients with lung fibrosis [6].

SP-D, which belongs to the collecting subgroup of the C-type lectin superfamily, is produced by two types of nonciliated epithelial cells in the peripheral airway (alveolar type II cells and Clara cells) and is secreted into the alveolar space [11]. It functions at the air–liquid interface to reduce surface tension and thereby prevent alveolar collapse and atelectasis; it also plays important roles in the innate immune system of the lungs [12]. The serum concentration of SP-D can reflect the pathological changes of lungs affected by idiopathic pulmonary fibrosis (IPF) by migration from the air space into the bloodstream [11].

In addition to type II pneumocyte-derived biomarkers, MMPs can degrade extracellular matrix components and numerous nonmatrix proteins [13]. MMPs and their inhibitors, tissue inhibitors of MMPs, have been implicated in the pathogenesis of pulmonary fibrosis based upon the results of clinical studies that showed elevated levels of MMPs in both blood and/or lung samples, indicating that they can serve as potential diagnostic and prognostic markers of chronic lung fibrosis [14–17].

Several biomarkers, such as interleukin-1β, type IV collagen, tumor necrosis factor-α, neopterin, and proteomic profiling, have shown potential significance in patients with lung fibrosis [18–21]. However, no previous investigations have evaluated the diagnostic values of pneumocyte-derived biomarkers in various pneumoconioses. To clarify the potential diagnostic biomarkers for asbestosis and silicosis, we measured the serum concentrations of KL-6, SP-D, and MMP-2, -7, and -9 in discriminating patients with asbestosis and silicosis from dust-exposed workers (DEWs) without pneumoconiosis and healthy controls (HCs).

## Methods

A case–control study of patients with asbestosis, patients with silicosis, DEWs without pneumoconiosis, and HCs was conducted using a sandwich-type electrochemiluminescence immunoassay (ECLIA) or commercially available enzyme-linked immunosorbent assay (ELISA) to detect the serum concentrations of KL-6, SP-D, and MMP-2, -7, and -9. All participants underwent chest X-ray and/or chest high-resolution computed tomography (HRCT) and pulmonary function testing. The study was approved by the Institutional Ethics Committee for Human Research, Beijing Chao-Yang Hospital. Informed consent was obtained from all participants before blood sample collection.

### Patients’ characteristics

In total, 43 outpatients with newly diagnosed asbestosis, 45 with silicosis and 40 dust-exposed workers (DEWs) without pneumoconiosis were sequentially recruited from the Department of Occupational Medicine and Toxicology, Beijing Chao-Yang Hospital, during a 2-year period (January 2015 to December 2016). All patients were diagnosed according to the diagnostic criteria of pneumoconiosis based on the 2011 International Labour Organization classification [22]. Patients with bronchial asthma, tuberculosis, autoimmune disease, severe liver and kidney dysfunction, and malignant tumors were excluded.

Forty DEWs, including 19 coal mine workers, 7 sand moulding workers, 5 jade polishers, 4 asbestos textile workers, and 5 boiler maintenance workers, had occupational exposure to silica or asbestos dust. They underwent all examinations without showing evidence of a pneumoconiosis.

The HCs comprised 45 age-, sex-, and smoking status-matched healthy volunteers from the health examination center of Beijing Chao-yang Hospital during the same period of time.

All participants’ smoking status was carefully determined, and they were categorized as non-smokers, ex-smokers (had quit smoking ≥12 months previously), and smokers (currently smoking or had quit smoking <12 months previously). Cigarette smoking is shown by pack-years.

### Occupational dust exposure

All participants completed a standardized questionnaire to collect information on their work history. All jobs within the participant’s working life were taken into account. The 43 patients with asbestosis were local residents who had been exposed to chrysotile dust or fibers, including 36 (83.7%) involved in the manufacture of asbestos textiles or asbestos-based products and 7 (16.3%) exposed to asbestos products in a working atmosphere (e.g., heat insulation workers and boiler maintenance workers). The 45 patients with silicosis were local residents who had been exposed to silica dust in the processing of jade. The asbestos product plants were open from the 1950s to 1970s, and the jade-processing factories were open from the 1970s to 1990s. Our hospital is the largest center for evaluation of occupational diseases in the city and is located 20 to 30 km away from the plants.

Because of the absence of atmospheric measurements and the lack of detailed information on the frequency of exposure for each job suspected to be associated with asbestos exposure, the duration of asbestos exposure (number of years) was determined.

### Measurements of KL-6, SP-D, and MMP-2, -7, and -9 concentrations

The serum samples were collected from each participant and stored at −80°C until serum analysis. All samples were measured within two weeks of the storage. The serum KL-6 concentration was measured by ECLIA using a Lumipulse G1200 Analyzer (Rebio, Fuji, Japan). The serum SP-D and MMP-2, -7, and -9 concentrations were measured by commercially available ELISA kits (RayBiotech, Norcross, GA, USA).

### Pulmonary function tests

Pulmonary function tests were performed according to the guidelines of the hospital physiology laboratory. Parameters used for analysis of the flow–volume curve were the forced vital capacity (FVC), forced expired volume in the first second (FEV_1_), and FEV_1_/FVC ratio. Each participant also underwent evaluation of their total lung capacity and diffusing capacity of the lung for carbon monoxide (DL_CO_ SB) (single-breath method, with the values corrected for the present hemoglobin concentration). The results are expressed as percentages of predicted values on the basis of age, height, and sex using equations established by the European Respiratory Society [23]. The forced expiratory maneuvers were repeated until three sequential measurements were obtained. The indices were obtained from the best curve, which was associated with the highest value of FEV_1_ plus FVC.

### Lung fibrosis scores on HRCT

HRCT scans in the patients with asbestosis were performed with 0.625-mm sections, a 1-second scan time, and a 10-mm interval in the apex base scans with inclusion of both lungs in the field of view. The HRCT images were reviewed by two experts blinded to the patients’ clinical data. Two experts scored the HRCT images, which comprised three images taken at the level of the aortic arch, carina, and 1 cm above the diaphragm. Each lung lobe was scored on a scale of 0 to 5 for interstitial abnormalities according to a previously described protocol [24]. The HRCT score for each patient was the sum of the five lung lobes and ranged from 1 to 25. The final score was the mean of the scores from the two experts. The interobserver correlation was good. The kappa coefficient for the correlation between the patterns was 0.742 (*P*=0.006).

Conventional chest radiographs were performed in each patient with silicosis and independently evaluated by two occupational medicine experts according to the International Labour Organization classification [22]. Briefly, the lung fields were divided into six zones on the posterior chest radiograph. When the highest density of small opacities was ≥1/0 and the distribution affected two or more zones, the patients were classified into Stage I. When the highest density of small opacities was ≥2/1 and the distribution affected more than four zones, or the highest density of small opacities was ≥3/2 and the distribution affected four or more zones, the patients were classified into Stage II. When the highest density of small opacities was ≥3/2 and the distribution affected four or more zones with aggregation of small or large opacities, or the diameter of the largest opacity was ≥20 × 10 mm, the patients were classified into Stage III. The interobserver correlation was good. The kappa coefficient for the correlation between the patterns was 0.653 (*P*=0.009).

### Statistical analysis

The results are expressed as mean ± standard deviation, and the differences among the four groups were tested using the Kruskal–Wallis test or one-way analysis of variance. Counting data were analyzed using the chi-square test. Correlations between parameters were assessed by Pearson’s correlation coefficient. The levels of serum biomarkers were further analyzed by a receiver operating characteristic (ROC) curve to determine the cut-off levels that resulted in the optimal diagnostic accuracy for each marker between the patients and controls. The use of these cut-off levels allowed for calculation of the sensitivity, specificity, and likelihood ratio of the biomarkers for separating the patients from the controls. Statistical analyses were performed using SPSS version 17.0 for Windows (SPSS Inc., Chicago, IL, USA). A *P* value of <0.05 was considered statistically significant.

## Results

### Demographics of the participants

The demographics of each participant group are summarized in Table 1. There was no significant difference in sex, age, or smoking status among the groups. However, the pulmonary function values, including the FEV_1_/FVC ratio, the predicted percentages of FEV_1_ and DL_CO_, showed significant differences among the groups.

**Table 1:** Demographics of the study participants

	Asbestosis	Silicosis	DEWs	HCs	*P* value
n	43	45	40	45	
Male: Female	19:24	23:22	21:19	22:23	0.879
Age, years	68.2 ± 8.6	65.1 ± 11.3	63.1 ± 8.7	65.6 ± 11.4	0.152
Smokers: nonsmokers	17:26	20:25	22:18	18:27	0.460
Current smoker: Exsmoker	6:11	8:12	13:9	11:7	0.275
Duration of exposure, years	7.4 ± 2.6	9.3 ± 3.1	7.8 ± 3.0	NA	0.107
FVC, predicted %	77.7 ± 23.4	79.2 ± 20.9	82.8 ± 18.2	83.3 ± 17.4	0.484
FEV_1_, predicted %	71.2 ± 12.5	77.1 ± 12.1	80.3 ± 11.5	80.8 ± 11.4	**0.002**
FEV_1_/FVC, %	75.5 ± 9.2	81.2 ± 8.0	81.7 ± 7.8	83.9 ± 11.7	**<0.001**
DL_CO_, predicted %	65.9 ± 14.1	74.3 ± 11.6	80.8 ± 7.8	80.6 ± 8.9	**<0.001**
Lung fibrosis scores	10.1 ± 6.4	NA	NA	NA	

*P* values were computed by chi-square test for sex and smoking status, and the one-way analysis of variance for age, duration of exposure and pulmonary function parameters. Significant results are highlighted in bold. *NA* not available

### Serum concentrations of KL-6, SP-D, and MMP-2, -7, and -9

**Fig. 1:**
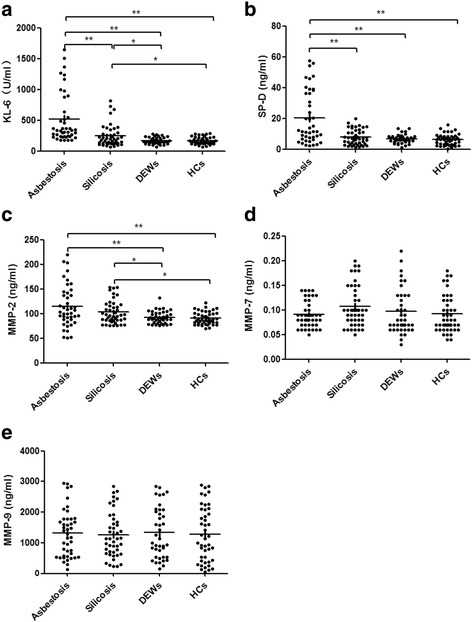
Comparison of serum concentrations of KL-6, SP-D, and MMP-2, -7, and -9 among the study groups using the Kruskal–Wallis test. **a**. KL-6. **b**. SP-D. **c**. MMP-2. **d**. MMP-7. **e**. MMP-9. *P<0.05; **P<0.01

As shown in Fig. 1, the serum KL-6 and MMP-2 concentrations were highest in patients with asbestosis and second highest in patients with silicosis; these concentrations were significantly different from those in DEWs and HCs (*P*<0.05). The serum SP-D concentration was significantly higher in patients with asbestosis than in patients with silicosis, DEWs, or HCs (*P*<0.01), whereas no significant difference was found among patients with silicosis, DEWs, and HCs. No significant differences were found in the serum MMP-7 or -9 concentration among patients with asbestosis, patients with silicosis, DEWs, and HCs. The serum concentrations of KL-6, SP-D, and MMP-2 were not significantly different among the various stages of silicosis (Table 2).

**Table 2:** Serum concentrations of of KL-6, SP-D and MMP-2 in the patients with various stages of silicosis

	Stage I	stage II	Stage III	*P* value
n	21	8	16	
KL-6, U/ml	200.2 ± 101.6	206.0 ± 127.8	314.6 ± 248.2	0.122
SP-D, ng/ml	7.0 ± 4.2	10.5 ± 7.0	7.3 ± 4.7	0.235
MMP-2, ng/ml	105.0 ± 27.4	100.9 ± 18.4	101.5 ± 19.3	0.869

*P* value were computed by Kruskal–Wallis test

### Correlations between the KL-6, SP-D, and MMP-2 concentrations and clinical parameters in patients with asbestosis

**Fig. 2:**
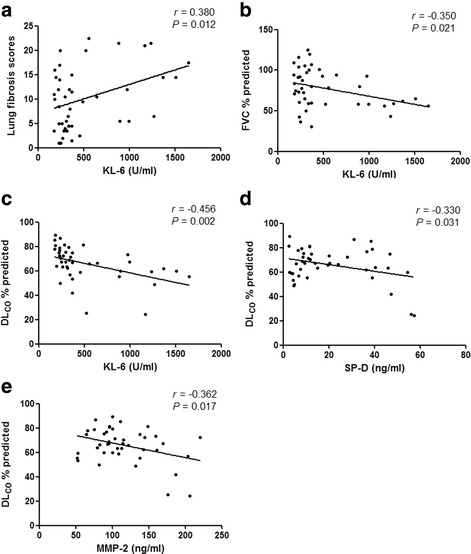
Correlation between KL-6, SP-D, and MMP-2 and clinical parameters in patients with asbestosis. **a**. KL-6 and lung fibrosis scores. **b**. KL-6 and FVC % predicted. **c**. KL-6 and DL_CO_ % predicted. **d**. SP-D and DL_CO_ % predicted. **e**. MMP-2 and DL_CO_ % predicted

As shown in Fig. 2, the serum KL-6 concentration was positively correlated with the lung fibrosis scores on HRCT in the patients with asbestosis while negatively correlated with the FVC % predicted and DL_CO_ % predicted (*P*<0.05). The serum SP-D and MMP-2 concentration was also negatively correlated with the DL_CO_ % predicted (*P*<0.05).

### ROC curve analysis for identification of asbestosis or asbestosis and silicosis

**Fig. 3:**
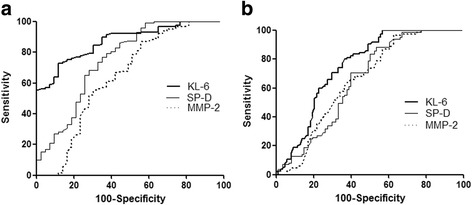
**a**. ROC curve analysis to differentiate patients with asbestosis from patients with silicosis, DEWs and HCs. **b**. ROC curve analysis to differentiate patients with asbestosis and silicosis from DEWs and HCs

ROC curve analysis was used to evaluate the ability of the serum KL-6, SP-D, and MMP-2 concentrations to differentiate patients with asbestosis from patients with silicosis, DEWs and HCs or asbestosis and silicosis from DEWs and HCs (Table 3). The area under the curve (AUC) for KL-6 was larger than that for SP-D and MMP-2 (Fig. 3), indicating that the order of diagnostic accuracy by the ROC curve was KL-6, SP-D, and MMP-2 in patients with asbestosis alone or in both patients with asbestosis and those with silicosis. The sensitivity and specificity of the combination of all three biomarkers for the diagnosis of asbestosis were 93% and 57%, respectively; however, they were 83% and 62% for the diagnosis of both asbestosis and silicosis.

**Table 3:** Cut-off values and the identifying ability of KL-6, SP-D and MMP-2 by ROC curve analysis

	Identifying asbestosis			Identifying asbestosis and silicosis		
	KL-6	SP-D	MMP-2	KL-6	SP-D	MMP-2
AUC	0.874	0.757	0.641	0.751	0.657	0.655
95% CI	0.815-0.919	0.686-0.818	0.564-0.712	0.679-0.813	0.581-0.728	0.579-0.725
Cut-off value	216 U/ml	8.82 ng/ml	110.6 ng/ml	222 U/ml	9.9 ng/ml	110.6 ng/ml
Sensitivity, %	88.4	65.1	44.2	62.5	46.6	37.5
Specificity, %	73.1	76.9	86.2	81.2	88.2	95.3
Likelihood ratio	3.28	2.82	3.19	3.32	3.96	7.97

## Discussion

In the present study, we showed that the diagnostic value of the serum biomarkers KL-6, SP-D, and MMP-2 was higher in patients with asbestosis or patients with silicosis than in DEWs and HCs. In patients with asbestosis, the serum KL-6 concentration was significantly correlated with the lung fibrosis scores on HRCT while negatively correlated with the FVC % predicted and DL_CO_ % predicted. In addition, the serum SP-D and MMP-2 concentrations were negatively correlated with the DL_CO_ % predicted in patients with asbestosis. The order of diagnostic accuracy according to the ROC curve was KL-6, SP-D, and MMP-2 in patients with asbestosis alone and in patients with asbestosis plus those with silicosis. The combination of KL-6, SP-D, and MMP-2 may improve the diagnostic sensitivity for asbestosis and silicosis.

KL-6 is mainly localized in the cytoplasm and membrane of alveolar type II epithelial cells and bronchial epithelial cells [7]. KL-6 is also expressed in the cytoplasm of Clara cells and in bronchial glands [7]. KL-6 can promote the proliferation and migration of fibroblasts, inhibit cell apoptosis, and increase the development of pulmonary fibrosis [25]. The proliferation of type II alveolar epithelial cells in dust-related lung fibroses such as asbestosis or silicosis may release KL-6 into the peripheral blood, resulting in a significantly elevated serum KL-6 concentration. An increased concentration of KL-6 in the epithelial lining fluid may stimulate fibrotic processes in patients with interstitial lung diseases and raise the possibility of the need for treatment with anti-KL-6 antibodies [26]. The serum KL-6 concentration in patients with interstitial lung diseases reflects the overall extent of interstitial lesions and serves as a powerful predictor of acute exacerbations of IPF [27, 28]. In the present study, KL-6 was higher with diagnostic value in both patients with asbestosis and patients with silicosis than in DEWs and HCs.

SP-D is also a type II pneumocyte-derived biomarker. SP-D plays an important role in lung innate immunity [29], and its serum concentration reflects the severity and prognosis of interstitial lung diseases [30, 31]**.** SP-A and SP-D are very similar in terms of structure and function. However, evaluation of their hydrophilicity suggests that SP-D migrates into the blood more easily than does SP-A in patients with IPF [11]**.** SP-D may more easily leak into the bloodstream, whereas SP-A remains bound to surfactant lipids in the alveolar space. Previous data have suggested that the serum SP-D concentration may more accurately reflect pathological changes in IPF-affected lungs than the serum SP-A concentration [11]. The present data show that the SP-D concentrations in patients with asbestosis were significantly higher than those in patients with silicosis, DEWs, or HCs. Our results indicate that the serum SP-D concentration might be an effective biomarker of asbestosis.

MMPs are a family of endopeptidases that can degrade several components of the extracellular matrix and control the activity of several proteins that function in immunity; damage, repair, and remodeling of collagen; and fibrosis [13]. The levels of MMP-7 and MMP-9 in both serum and bronchoalveolar lavage fluid are significantly higher in patients with IPF than in healthy controls [32]. MMP-2, -7, and -9 have also been shown to be upregulated in the lungs in patients with IPF, particularly in alveolar macrophages, hyperplastic epithelial cells, and myofibroblasts [14, 32, 33]. Elevated serum MMP-7 concentrations are correlated with severe lung fibrosis and poorer survival in patients with IPF [34]. Our data failed to show the diagnostic value of the serum MMP-7 and -9 concentrations in patients with asbestosis and in those with silicosis compared with DEWs and HCs, but our data did show the diagnostic value of the MMP-2 concentration. This finding may suggest that MMPs have different roles between dust-related fibrotic lung diseases and IPF.

The typical radiological abnormalities associated with asbestosis are pulmonary fibrosis and pleural plaques [35]. Like other pulmonary fibrotic diseases, asbestosis can be evaluated by the lung fibrosis scores on HRCT to describe the degree of pulmonary fibrosis [36]. As in patients with IPF, our data showed that the serum KL-6 concentration was correlated with the extent of diffuse fibrosis in patients with asbestosis; however, it was not correlated with the density in patients with nodular fibrosis. The serum KL-6 concentration was significantly correlated with lung fibrosis scores on HRCT, indicating the use of KL-6 in the evaluation of pulmonary fibrosis. In the analysis of the KL-6, SP-D, and MMP-2 concentrations and pulmonary function in patients with asbestosis, a higher serum KL-6 concentration was significantly negatively correlated with the FVC % predicted and DL_CO_ % predicted, suggesting that high expression of KL-6 is associated with decreased pulmonary ventilation function and diffusion function in patients with asbestosis. The serum SP-D and MMP-2 concentrations were negatively correlated with DL_CO_ % predicted, indicating impairment of pulmonary diffusion function [37, 38]. The ROC curve analysis showed that the area under the curve of KL-6 was the largest, indicating that it is more effective than SP-D and MMP-2 in the diagnosis of asbestosis or silicosis. The sensitivity of the combination of all three biomarkers was higher than that of any single serum marker. Therefore, the combination of KL-6, SP-D, and MMP-2 can improve the sensitivity of diagnosis of asbestosis and silicosis.

Some limitations of this study should be mentioned. First, we could not continuously monitor the biomarker concentrations in every patient to evaluate the dynamic changes in the progression of asbestosis and silicosis. In addition, other biomarkers may be effective for monitoring and evaluating disease progression and prognosis.

## Conclusions

This study showed that KL-6, SP-D, and MMP-2 are readily available biomarkers for the adjuvant diagnosis of asbestosis and silicosis. The combination of all three biomarkers may improve the sensitivity of differentiating patients with asbestosis or silicosis from DEWs or HCs. Further study is warranted to justify KL-6, SP-D and MMP-2 as confirmation of early occupational lung fibrosis in the workers exposure to dusts for the possibility of intervention or prevention of the diseases.

## Abbreviations

AUC: The area under the curve
DEWs: Dust-exposed workers
DL_CO_: Diffusing capacity of the lung for carbon monoxide
ECLIA: Electrochemiluminescence immunoassay
ELISA: Enzyme-linked immunosorbent assay
FEV_1_: Forced expired volume in the first second
FVC: Forced vital capacity
HCs: Healthy controls
HRCT: High-resolution computed tomography
IPF: Idiopathic pulmonary fibrosis
KL-6: Krebs von den Lungen-6
MMP: Matrix metalloproteinase
ROC: Receiver operating characteristic
SP-D: Surfactant protein D
